# Pharmacokinetic/pharmacodynamic (PK/PD) simulation for dosage optimization of colistin and sitafloxacin, alone and in combination, against carbapenem-, multidrug-, and colistin-resistant *Acinetobacter baumannii*

**DOI:** 10.3389/fmicb.2023.1275909

**Published:** 2023-11-30

**Authors:** Vipavee Rodjun, Preecha Montakantikul, Jantana Houngsaitong, Kamonchanok Jitaree, Wichit Nosoongnoen

**Affiliations:** ^1^Faculty of Pharmacy, Siam University, Bangkok, Thailand; ^2^Division of Clinical Pharmacy, Department of Pharmacy, Faculty of Pharmacy, Mahidol University, Bangkok, Thailand; ^3^Division of Pharmacy Practice, Faculty of Pharmaceutical Sciences, Ubon Ratchathani University, Ubon Ratchathani, Thailand

**Keywords:** colistin, sitafloxacin, *Acinetobacter baumannii*, Monte Carlo simulation, multidrug-resistant *Acinetobacter baumannii*, carbapenem-resistant *Acinetobacter baumannii*, combination

## Abstract

To the best of our knowledge, to date, no study has investigated the optimal dosage regimens of either colistin or sitafloxacin against drug-resistant *Acinetobacter baumannii* (*A. baumannii*) infections by using specific parameters. In the current study, we aimed to explore the optimal dosage regimens of colistin and sitafloxacin, either in monotherapy or in combination therapy, for the treatment of carbapenem-, multidrug-, and colistin-resistant *A. baumannii* infections. A Monte Carlo simulation was applied to determine the dosage regimen that could achieve the optimal probability of target attainment (PTA) and cumulative fraction of response (CFR) (≥90%) based on the specific parameters of each agent and the minimal inhibitory concentration (MIC) of the clinical isolates. This study explored the dosage regimen of 90, 50, 30, and 10 mL/min for patients with creatinine clearance (CrCL). We also explored the dosage regimen for each patient with CrCL using combination therapy because there is a higher possibility of reaching the desired PTA or CFR. Focusing on the MIC90 of each agent in combination therapy, the dosage regimen for colistin was a loading dose of 300 mg followed by a maintenance dose ranging from 50 mg every 48 h to 225 mg every 12 h and the dosage regimen for sitafloxacin was 325 mg every 48 h to 750 mg every 12 h. We concluded that a lower-than-usual dose of colistin based on specific pharmacokinetic data in combination with a higher-than-usual dose of sitafloxacin could be an option for the treatment of carbapenem-, multidrug-, and colistin-resistant. *A. baumannii.* The lower dose of colistin might show a low probability of adverse reaction, while the high dose of sitafloxacin should be considered. In the current study, we attempted to find if there is a strong possibility of drug selection against crucial drug-resistant pathogen infections in a situation where there is a lack of new antibiotics. However, further study is needed to confirm the results of this simulation study.

## Introduction

*Acinetobacter baumannii* (*A. baumannii*) is one of the most important gram-negative pathogens that cause various nosocomial infections (García-Garmendia et al., 2001; Cisneros and Rodríguez-Baño, 2002; Werarak et al., 2012; Sieniawski et al., 2013; Almasaudi, 2018; Centers for Disease Control and Prevention, n.d.). Drug-resistant *A. baumannii* infections, including multidrug-resistant *A. baumannii* (MDR-AB), carbapenem-resistant *A. baumannii* (CRAB), and colistin-resistant *A. baumannii* (CoR-AB) infections, are a crucial problem because they cause prolonged hospitalization and a high mortality rate (Sunenshine et al., 2007; Kaye and Pogue, 2015). The study by Appaneal et al. showed that the inpatient mortality rate was higher in those with MDR-AB than those with non-MDR-AB infection (aOR 1.61) and in those with CRAB than non-CRAB infection (aOR 1.68). A hospitalization duration of more than 10 days was higher in those with MDR-AB compared to those with non-MDR-AB infection and in those with CRAB compared to those with non-CRAB infection (Appaneal et al., 2022). Moreover, the clinical outcomes were worse among patients with MDR-AB and/or CRAB infections (Appaneal et al., 2022). Similarly, the meta-analysis showed that the CRAB could increase the risk of high mortality rate in patients (Lemos et al., 2014). Compared to colistin-susceptible AB infection, patients with CoR-AB bloodstream infection had higher mortality (100% vs. 50%, respectively (*p* = 0.001)) and died sooner (*p* = 0.006) (Papathanakos et al., 2020).

Colistin has gained attention for its use in the treatment of drug-resistant *A. baumannii* infections (Falagas et al., 2010; Lim et al., 2011; Batirel et al., 2014; Kalin et al., 2014; López-Cortés et al., 2014; Yilmaz et al., 2015; Amat et al., 2018; Liang et al., 2018; Dickstein et al., 2019). The optimal dosage regimens for colistin have been investigated from the past to the present based on the population pharmacokinetic model and pharmacokinetic/pharmacodynamic (PK/PD) index of colistin (Garonzik et al., 2011; Nation et al., 2017). The usual PK/PD index of colistin is the average steady-state plasma colistin concentration (C_ss,avg_). However, recent studies found that the most predictive PK/PD index of colistin against *A. baumannii* was the ratio of the area under the unbound concentration-time curve to the minimum inhibitory concentration (ƒAUC/MIC) (Dudhani et al., 2010; Cheah et al., 2015). A common adverse drug reaction from colistin is nephrotoxicity, which occurs in a dose- and time-dependent manner (Spapen et al., 2011; Dai et al., 2014). Therefore, the challenge of exploring a colistin dosage regimen is focused on both increasing efficacy and lowering toxicity.

Sitafloxacin is a fluoroquinolone that has shown excellent *in vitro* activity against drug-resistant *A. baumannii* (Dong et al., 2015; Huang et al., 2015; Rodjun et al., 2020). Its population pharmacokinetic model and PK/PD index have also been studied (Tanigawara et al., 2013). A key feature of sitafloxacin is its excellent penetration into the epithelial lining fluid (ELF) of critically ill patients with pneumonia; according to the data of Paiboonvong et al., the AUC0–8 h of ELF/unbound plasma ratio was 0.85 (Paiboonvong et al., 2019). Moreover, sitafloxacin in combination with colistin can decrease the MIC values of either colistin or sitafloxacin. This *in vitro* activity was observed when using this combination against extensively drug-resistant *A. baumannii* (XDR-AB) (Dong et al., 2015), MDR-AB, CRAB, and CoR-AB (Rodjun et al., 2020).

The World Health Organization (WHO) announced that there are declining private investments and a lack of innovation in the development of new antibiotics. Over 30 antibiotics are still in the clinical development pipeline (Kmietowicz, 2017; Butler et al., 2022). Because of the lack of novel antibiotics to treat drug-resistant bacterial infections, the standard guideline for treating drug-resistant pathogen infections still recommends using familiar antibiotics or some regimens that use the combination therapy (Tamma et al., 2023). The combination regimens of colistin with other antibiotics such as sulbactam, tigecycline, and carbapenems have been options for the management of drug-resistant *A. baumannii* infections (Garonzik et al., 2011; Lim et al., 2011; Batirel et al., 2014; Kalin et al., 2014; López-Cortés et al., 2014; Amat et al., 2018; Dickstein et al., 2019). From a previous *in vitro* study with sitafloxacin, the combination of colistin and sitafloxacin is one interesting possibility. This study aimed to explore the optimal dosage regimens of colistin and sitafloxacin, either in monotherapy or in combination therapy, for the treatment of MDR-AB, CRAB, and CoR-AB infections using a Monte Carlo simulation that was based on the specific population pharmacokinetics and PK/PD index of each agent.

## Materials and methods

### Microbiology

Data on *A. baumannii* were obtained from a prior study by Rodjun et al. (2020). Three hundred *A. baumannii* clinical isolates were comprised of MDR-AB–263 isolates (87.7%), CRAB–258 isolates (86%), and CoR-AB–43 isolates (14.3%). The MIC50/90 of colistin in MDR-AB and CRAB was 2/4 mg/L and that of CoR-AB was 8/8 mg/L. The MIC50/90 of sitafloxacin in MDR-AB and CRAB was 1/2 mg/L and that of CoR-AB was 0.5/1 mg/L. The MIC50/90 of colistin in combination regimens in MDR-AB and CRAB was 0.5/1 mg/L and that of CoR-AB was 1/2 mg/L. The MIC50/90 of sitafloxacin in combination regimens in MDR-AB and CRAB was 0.5/1 mg/L and that of CoR-AB was 0.25/1 mg/L.

### Pharmacokinetic model

#### Colistin

The population pharmacokinetic models for colistimethate sodium (CMS) and colistin were two-compartment and one-compartment models, respectively. We used pharmacokinetic data from Nation et al. (2017), who studied the dosing guidance for colistin in critically ill patients with a CrCL of 0–236 mL/min. The parameters were randomly generated for each estimated mean, and the %IIV of the parameters are shown in Table 1. The equations below were modified according to the study of Garonzik et al. (2011) and represent the differential equations for the disposal of CMS and colistin. The unbound fraction of 0.49 +/− 0.11, determined by ultracentrifugation in samples collected from the patients in the prior study, was used (Nation et al., 2017).

(1) $$\frac{\mathrm{dCMSc}}{dt}=R1-CLD1\times \left(\frac{\mathrm{CMSc}}{V1}-\frac{\mathrm{CMSp}}{V2}\right)-\left(CLT_{CMS}\times \frac{\mathrm{CMSc}}{V1}\right)$$

(2) $$\frac{\mathrm{dCMSp}}{dt}=CLD1\times \left(\frac{\mathrm{CMSc}}{V1}-\frac{\mathrm{CMSp}}{V2}\right)$$

(3) $$\frac{\mathrm{dColistin}}{dt}=CLNR_{CMS}\times \frac{\mathrm{CMSc}}{V1}-\left(CLT_{C}\times \frac{\mathrm{Colistin}}{V3}\right)$$

**Table 1:** Population pharmacokinetic parameters of colistin (Nation et al., 2017).

Agent	Parameter	Unit	Estimate	%SE	%IIV
CMS	V1	L	12.9	-	40.4
	V2	L	16.1	-	70.9
	CLD1	L	9.57	10.5	80.1
	CLR	L/h/CrCL	0.0340	6.85	75.2
	CLNR_CMS_	L/h	2.52	3.71	39.8
Colistin	V3	L	57.2	5.13	43.5
	CLT_C_	L/h	3.59	-	37.9
	CLR_C_	L/h/CrCL	0.00834	27.7	-
	CLNR_C_	L/h	3.11	4.38	-

V1, Central volume for CMS, V2, Peripheral volume for CMS, CLD1, Distributional clearance between the central and peripheral compartments for CMS, CLR, Renal clearance of CMS, CLNRCMS, Non-renal clearance of CMS, V3, volume of distribution of formed colistin, CLTC, Total clearance of colistin, CLRC, Renal clearance of colistin, CLNRC, Non-renal clearance of colistin, %SE, standard error or the precision of the estimates, %IIV, inter-individual variability in the population (standard deviation, SD, were calculated from %IVV x mean), CrCL, creatinine clearance, CMSC, mass of CMS in the central compartment, CMSp, mass of CMS in the peripheral compartment, colistin, mass of colistin in the single compartment, R1, infusion rate of CMS, CLTCMS, total intrinsic clearance for CMS.

#### Sitafloxacin

The population pharmacokinetic model for oral sitafloxacin was assumed to follow the one-compartment model with first-order absorption (Tanigawara et al., 2013). We used pharmacokinetic data from Tanigawara et al. (2013), who used clinical data from clinical pharmacology studies, including a study on healthy, elderly but renally impaired patients (Nakashima et al., 1995; Nakashima, 2008; Nakashima and Kawada, 2008; Sekino, 2008), and the clinical PK/PD study on patients with respiratory tract infections (Saito et al., 2008). The parameters are shown in Table 2. The equation below was used to calculate the plasma sitafloxacin concentration (Jambhekar and Breen, 2012). An unbound fraction of sitafloxacin of 0.388 was used (Tanigawara et al., 2013).

(4) $$\frac{dX}{dt}=K_{a}X_{a}-KX\text{,}$$

**Table 2:** Population pharmacokinetic parameters of sitafloxacin (Tanigawara et al., 2013).

Agent	Parameter	Unit	Estimate	$\omega^{2}$
Sitafloxacin	CL_t_/F	L	2.58 x CrCL	0.0757
	V/F	L/Kg	1.72	0.087
	k_a_	h^−1^	1.67	4.57

CLt, total clearance, V, volume of distribution (age < 65 years), ka, absorption constant in a fasted state, ω 2, the variance of the estimated value (SD were calculated from the square root of ω 2 × 100% × estimate).

where dX/dt = the rate of change of the amount of drug in the plasma, X = the mass of drug in the plasma at time t, X_a_ = the mass of absorbable drug at time t, K_a_ and K = the first-order of absorption and elimination rate constants, respectively, K_a_X_a_ = the first-order rate of absorption, and KX = the first-order rate of elimination.

### Pharmacokinetic or pharmacodynamic index

#### Colistin

The pharmacokinetic/pharmacodynamic index (PK/PD) index of colistin is characterized by ƒAUC/MIC ≥7.4, which showed a 2-log_10_ reduction of MDR *A. baumannii* clinical isolate strain 248-01-C.248 (MIC of colistin is 1 mg/L) in a mouse model with a thigh infection. This value resulted in bacterial burdens in mouse thighs determined at 2 h after inoculation (untreated controls) and 24 h later (untreated controls and colistin-treated subjects) (Cheah et al., 2015). The model with the thigh infection has been used in most *in vivo* studies (Craig, 1998) and is considered to be the gold standard for evaluating the efficacy of antimicrobials since its high degree of translation to human patients (Yedle et al., 2023). Moreover, the thigh infection model is considered to be an adequate simulator because the model allows for the dissemination of the offending pathogen in blood and viscera to occur as in the clinical status of bacteremia (Pantopoulou et al., 2007).

#### Sitafloxacin

The PK/PD index of sitafloxain is characterized by ƒAUC/MIC >30, which showed an eradication effect of 96.4% on respiratory tract infection (RTIs) isolates. This value was determined for individual PK parameters with MIC in 91 RTI isolates, and the attainment rates of the ƒAUC/MIC were calculated (Tanigawara et al., 2013).

### Simulated dosage regimens

#### Colistin

The dosage regimens were chosen according to the study of Nation et al. (2017), the guidelines of the European Medicine Agency (EMA) (Nation et al., 2016) and the United States Food and Drug Administration (FDA) (Nation et al., 2016), and the recommended dosage regimens by Siriraj Hospital, Thailand, and our study’s dosage regimens. The creatinine clearance (CrCL) values used for the simulation were 90, 50, 30, and 10 mL/min. Each dose was infused for 30 min, and each dosage regimen starts with the loading dose (LD) of 450 mg or 300 mg. The maintenance doses vary from 50 mg every 48 h to 450 mg every 12 h according to the CrCL value.

#### Sitafloxacin

The dosage regimens were chosen according to the manufacturer’s recommendations (Pharmaceuticals and Medical Devices Agency, n.d.) and our study’s dosage regimens. The regimens were administered orally to an inpatient in a fasted state who weighed 60 kg and was under 65 years of age. The CrCL values used in this study were 90, 50, 30, and 10 mL/min. The doses vary from 50 mg every 48 h to 1,500 mg every 12 h according to the CrCL value.

### Monte Carlo simulation

A Monte Carlo Simulation (Crystal Ball version 2017; Decisioneering Inc., Denver, CO United States) was applied to generate 10,000 subjects for each regimen. Log-normal distributions were studied for between-patient variability of each parameter except the unbound fraction of colistin, which was studied by the uniform distribution. The probability of target attainment (PTA) was determined as the percentage of all 10,000 estimates that achieved or exceeded the pharmacodynamic surrogate indices of each agent. Both colistin and sitafloxacin use ƒAUC/MIC. The AUC was determined using the linear trapezoidal rule, while ƒ was the unbound fraction of each agent. The MIC values were calculated from a prior study (Rodjun et al., 2020). The cumulative fraction of response (CFR) was calculated as the proportion of %PTA of each MIC according to the MIC distribution. The PTA and CFR (Asuphon et al., 2016; Jitaree et al., 2019; Leelawattanachai et al., 2020), which we calculated at the steady state, were considered optimal at ≥90%. This study was approved by the Ethics Committee of the Faculty of Dentistry/Faculty of Pharmacy, Mahidol University, Phutthamonthon District, Nakhon Pathom, Thailand (COE.No.MU-DT/PY-IRB 2020/001.1501).

## Result

### Colistin monotherapy

The dosage regimens of colistin that achieved a PTA of ≥90% for the MIC50 of MDR-AB and CRAB (2 mg/L) were the maintenance doses of 225 mg every 12 h, 150 mg every 24 h, 75 mg every 24 h, and 50 mg every 24 h for CrCL values of 90, 50, 30, and 10 mL/min, respectively. The dosage regimens that achieved a PTA of ≥90% for the MIC90 of MDR-AB and CRAB (4 mg/L) were maintenance doses of 300 mg every 12 h, 150 mg every 24 h, and 75 mg every 24 h for CrCL values of 50, 30, 10 mL/min, respectively. The dosage regimens that achieved a PTA of ≥90% for the MIC50/90 of CoR-AB (8 mg/L) were maintenance doses of 450 mg every 12 h, 300 mg every 12 h, and 150 mg every 24 h for CrCL values 50, 30, and 10 mL/min, respectively. No dosage regimen was recommended for patients with CrCL 90 mL/min at MIC of 2 and 4 mg/L.

The dosage regimens of colistin that achieved a CFR of ≥90% for MDR-AB and CRAB were maintenance doses of 300 mg every 12 h, 150 mg every 12 h, 150 mg every 24 h, and 50 mg every 24 h for CrCL values of 90, 50, 30, and 10 mL/min, respectively. The maintenance doses of 450 mg every 12 h, 300 mg every 12 h, and 150 mg every 24 h were recommended for patients with CrCL values 50, 30, and 10 mL/min, respectively. No dosage regimen was recommended for patients with CrCL values of 90 mL/min.

As can be observed from the results, a lower CrCL value (50, 30, and 10 mL/min) can achieve the target by the usual dosage regimen including the USFDA recommended. Meanwhile, a CrCL value of 90 mL/min should be used in our regimen, which is higher than the usual dose. The results table of colistin monotherapy is shown in the Supplementary Table S1.

### Sitafloxacin monotherapy

The dosage regimens that achieved a PTA of ≥90% for the MIC50 of CoR-AB (0.5 mg/L) were doses of 375 mg every 12 h, 225 mg every 12 h, 250 mg every 24 h, and 175 mg every 48 h for CrCL values of 90, 50, 30, and 10 mL/min, respectively. The dosage regimen that achieved a PTA of ≥90% for the MIC50 of MDR-AB and CRAB, and the MIC90 of CoR-AB (1 mg/L) were doses of 750 mg every 12 h, 425 mg every 12 h, 500 mg every 24 h, and 325 mg every 48 h for CrCL values of 90, 50, 30, and 10 mL/min, respectively. The dosage regimens that achieved a PTA of ≥90% for the MIC90 of MDR-AB and CRAB (2 mg/L) were doses of 1,500 mg every 12 h, 750 mg every 12 h, 1,000 mg every 24 h, and 675 mg every 48 h for CrCL values of 90, 50, 30, and 10 mL/min, respectively.

The dosage regimens of sitafloxacin that achieved a CFR of ≥90% of MDR-AB and CRAB were doses of 1,500 mg every 12 h, 750 mg every 12 h, and 500 mg every 48 h for CrCL 90, 50, and 10 mL/min, respectively. For CrCL 30 mL/min, these doses were 800 mg every 24 h and 750 mg every 24 h of MDR-AB and CRAB, respectively. The dosage regimens for CoR-AB were 1,000 mg every 12 h, 500 mg every 12 h, 750 mg every 24 h, and 500 mg every 48 h for CrCL values of 90, 50, 30, and 10 mL/min, respectively.

As can be observed from the results, the manufacturer’s regimen cannot reach the target in any CrCL values. All the recommended dosage regimens were generated in this study. The lowest dose to achieve the target was 500 mg every 48 h. The results table of sitafloxacin monotherapy is shown in the Supplementary Table S2.

### Colistin in combinations

The dosage regimens of colistin in combinations that achieved a PTA of ≥90% for the MIC50 of MDR-AB and CRAB (0.5 mg/L) were maintenance doses of 100 mg every 24 h for CrCL values of 90 mL/min and 50 mg every 48 h for 50, 30, and 10 mL/min. The dosage regimens that achieved a PTA of ≥90% for the MIC90 of MDR-AB, CRAB, and the MIC50 of CoR-AB (1 mg/L) were maintenance doses of 150 mg every 12 h for CrCL 90 mL/min, 75 mg every 24 h for CrCL 50 mL/min, and 50 mg every 48 h for CrCL 30 and 10 mL/min. The dosage regimens for the MIC90 of CoR-AB were the same as those for the MIC50 of MDR-AB and CRAB in monotherapy.

The dosage regimens that achieved a CFR of ≥90% for MDR-AB and CRAB were maintenance doses of 100 mg every 12 h for CrCL 90 mL/min and 50 mg every 48 h for CrCL values of 50, 30, and 10 mL/min. The maintenance doses for CoR-AB were 180 mg every 12 h, 150 mg every 24 h, 75 mg every 24 h, and 50 mg every 48 h for CrCL values of 50, 30, and 10 mL/min, respectively.

Overall, the doses of colistin in combination were lower than in monotherapy. The lower CrCL values (50, 30, and 10 mL/min) can achieve the target by the usual regimen while the CrCL values of 90 mL/min should use a higher dose, especially at MIC 2 mg/L.

### Sitafloxacin in combinations

The dosage regimens of sitafloxacin in combinations that achieved a PTA of ≥90% for the MIC50 of CoR-AB (0.25 mg/L) were doses of 200 mg every 12 h, 125 mg every 12 h, 125 mg every 24 h, and 50 mg every 24 h for CrCL values of 90, 50, 30, and 10 mL/min, respectively. The dosage regimens that achieved a PTA of ≥90% for the MIC50 of MDR-AB and CRAB (0.5 mg/L) were the same as those for the MIC50 of CoR-AB in monotherapy. The dosage regimens that achieved a PTA of ≥90% for the MIC90 of MDR-AB and CRAB (1 mg/L) were the same as those for the MIC50 of MDR-AB and CRAB (1 mg/L) in monotherapy.

The optimal doses of sitafloxacin in combinations that achieved a CFR of ≥90% for MDR-AB and CRAB were doses of 750 mg every 12 h, 400 mg every 12 h, 500 mg every 24 h, and 300 mg every 48 h for CrCL values of 90, 50, 30, and 10 mL/min, respectively. The dosage regimens that achieved a CFR of ≥90% for CoR-AB were doses of 750 mg every 12 h, 400 mg every 12 h, 300 mg every 24 h, and 200 mg every 48 h for CrCL values of 90, 50, 30, and 10 mL/min, respectively.

The dosage regimens in combinations were lower than those in monotherapy. However, the manufacturer’s regimen cannot reach the target in any CrCL values.

**Figure 1:**
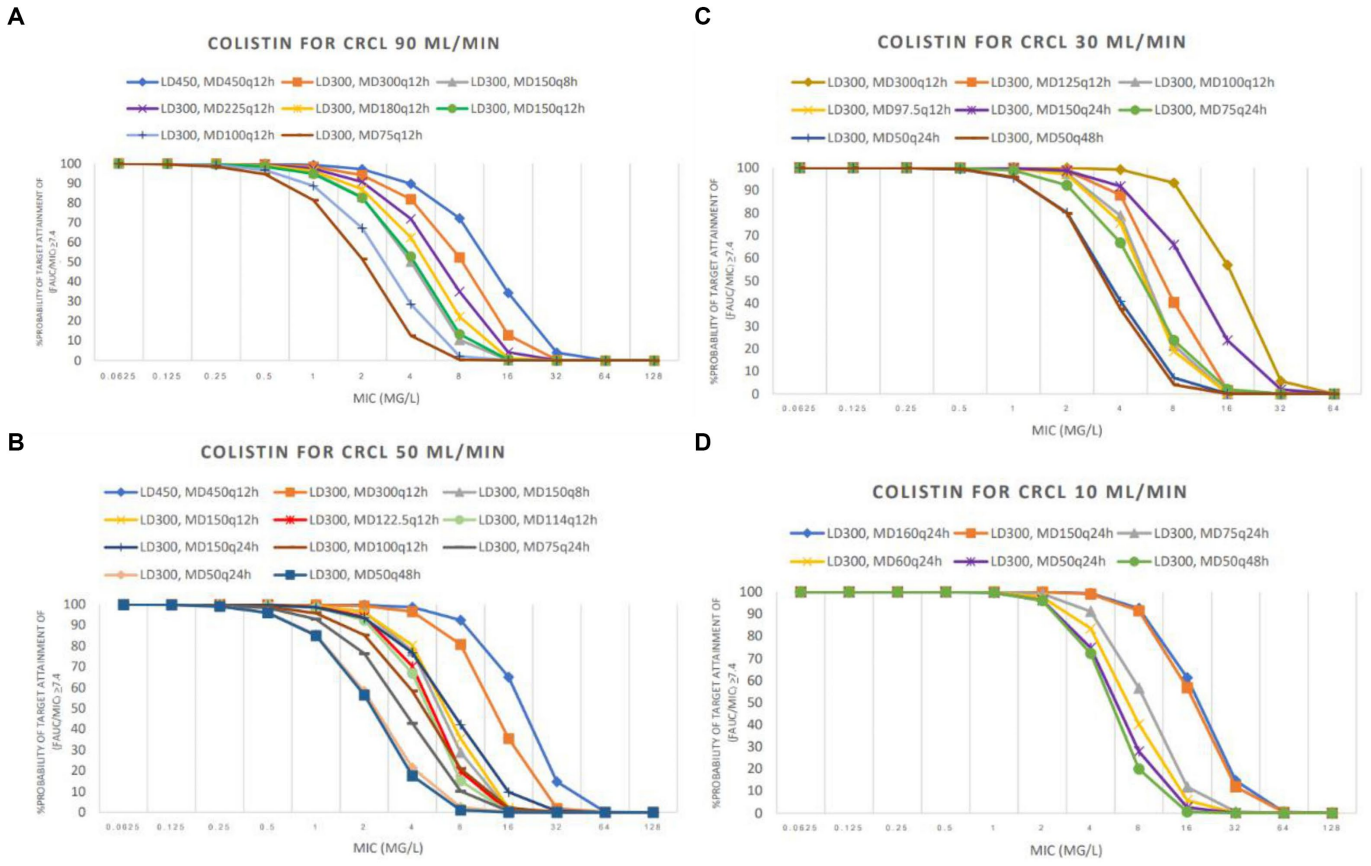
Probability of target attainment (PTA) achieved with colistin used in groups CrCL 90 mL/min **(A)**, CrCL 50 mL/min **(B)**, CrCL 30 mL/min **(C)**, and CrCL 10 mL/min **(D)**. LD: loading dose (in mg colistin base activity), MD: maintenance doses (in mg colistin base activity).

**Figure 2:**
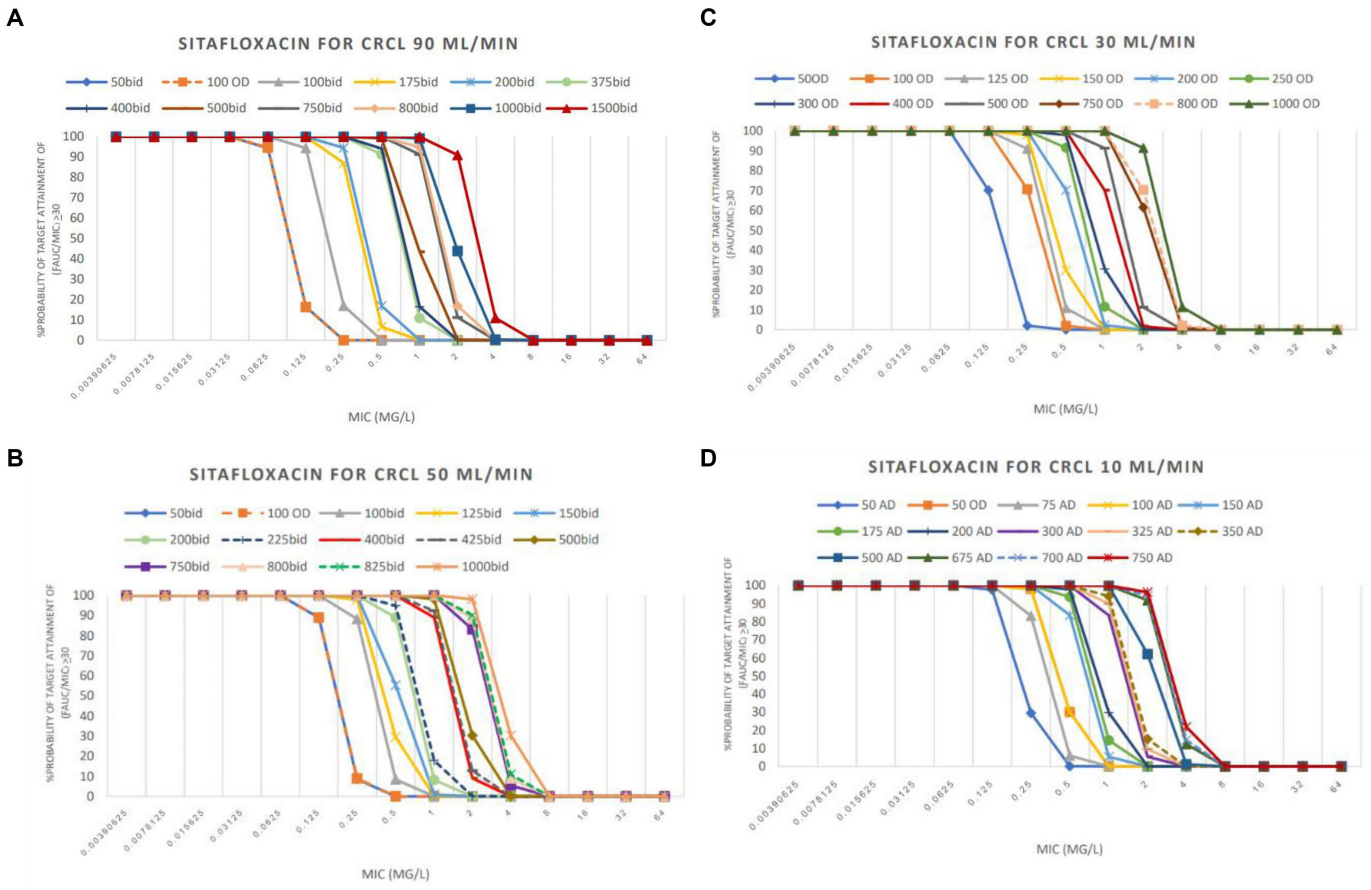
PTA achieved with sitafloxacin used in groups CrCL 90 mL/min **(A)**, CrCL 50 mL/min **(B)**, CrCL 30 mL/min **(C)**, and CrCL 10 mL/min **(D)**.

The PTA analyses of various colistin and sitafloxacin regimens and CrCL are shown in Figures 1, 2, respectively. The PTA and CFR of each dosage regimen are shown in Tables 3–5 and in Supplementary material.

**Table 3:** The lowest recommended dose of colistin-sitafloxacin: focus on the MIC_90_ of each agent in combination.

CrCL (mL/min)	MIC_90_ (mg/L)	
	Colistin: 1 (MDR-AB, CRAB) Sitafloxacin: 1	Colistin: 2 (CoR-AB) Sitafloxacin: 1
90 mL/min	Colistin: LD 300 mg, MD 150 mg q 12 h Sitafloxacin: 750 mg q 12 h	Colistin: LD 300 mg, MD 225 mg q 12 h Sitafloxacin: 750 mg q 12 h
50 mL/min	Colistin: LD 300 mg, MD 75 mg q 24 h Sitafloxacin: 425 mg q 12 h	Colistin: LD 300 mg, MD 150 mg q 24 h Sitafloxacin: 425 mg q 12 h
30 mL/min	Colistin: LD 300 mg, MD 50 mg q 48 h Sitafloxacin: 500 mg q 24 h	Colistin: LD 300 mg, MD 75 mg q 24 h Sitafloxacin: 500 mg q 24 h
10 mL/min	Colistin: LD 300 mg, MD 50 mg q 48 h Sitafloxacin: 325 mg q 48 h	Colistin: LD 300 mg, MD 50 mg q 24 h Sitafloxacin: 325 mg q 48 h

**Table 4:** %Probability of target attainment (PTA) and %Cumulative fraction of response (CFR) of the studied colistin regimens for each type of isolate in combinations.

Dosage regimen	Daily MD	%PTA of MDR-AB and CRAB		%CFR MDR-AB	%CFR CRAB	%PTA of CoR-AB		%CFR CoR-AB
		MIC_50_ (0.5 mg/L)	MIC_90_ (1 mg/L)			MIC_50_ (1 mg/L)	MIC_90_ (2 mg/L)	
**CrCL 90 mL/min** LD 300 mg, MD 150 mg q 8 h (Siriraj) LD 300 mg, MD 225 mg q12h (Our study) LD 300 mg, MD 180 mg q 12 h (Nation et al.) LD 300 mg, MD150 mg q 12 h (Siriraj, EMA, and FDA) LD 300 mg, MD 100 mg q 12 h (Our study) LD 300 mg, MD 100 mg q 24 h (Our study) LD 300 mg, MD 75 mg q 24 h (Our study)	450 mg 450 mg 360 mg 300 mg 200 mg 100 mg 75 mg	98.88 99.27 98.89 98.39 96.72 **93.26** 88.72	95.54 97.23 96.05 **94.29** 89.24 82.11 73.27	96.99 98.12 97.38 96.37 **93.40** 89.26 83.85	97.03 98.12 97.41 96.41 **93.47** 89.37 84.00	95.54 97.23 96.05 **94.29** 89.24 82.11 73.27	83.31 **90.62** 87.20 82.22 68.14 58.91 45.32	88.45 92.66 **90.37** 87.48 80.04 73.35 64.03
**CrCL 50 mL/min** LD 300 mg, MD 150 mg q 12 h (Siriraj, EMA, and FDA) LD 300 mg, MD 122.5 mg q 12 h (Nation et al.) LD 300 mg, MD 114 mg q 12 h (FDA) LD 300 mg, MD 150 mg q 24 h (Our study) LD 300 mg, MD 100 mg q 24 h (Our study) LD 300 mg, MD 75 mg q 24 h (Our study) LD 300 mg, MD 50 mg q 24 h (Our study) LD 300 mg, MD 50 mg q 48 h (Our study)	300 mg 245 mg 228 mg 150 mg 100 mg 75 mg 50 mg 25 mg	99.82 99.8 99.76 99.72 99.00 98.33 96.14 **95.97**	99.2 98.89 98.62 98.63 95.71 **92.91** 85.50 85.00	99.25 98.97 98.77 98.91 97.24 95.61 91.55 **91.23**	99.25 98.98 98.79 98.92 97.27 95.67 91.65 **91.33**	99.2 98.89 98.62 98.63 95.71 **92.91** 85.50 85.00	96.17 94.08 92.49 **93.48** 85.22 76.38 58.13 56.42	95.42 93.90 93.07 94.63 89.60 84.95 75.10 74.24
**CrCL 30 mL/min** LD 300 mg, MD 125 mg q 12 h (EMA) LD 300 mg, MD 100 mg q 12 h (Siriraj) LD 300 mg, MD 97.5 mg q 12 h (Nation et al.) LD 300 mg, MD 150 mg q 24 h (FDA) LD 300 mg, MD 75 mg q 24 h (Our study) LD 300 mg, MD 50 mg q 24 h (Our study) LD 300 mg, MD 50 mg q 48 h (Our study)	250 mg 200 mg 195 mg 150 mg 75 mg 50 mg 25 mg	99.98 99.96 99.98 99.96 99.83 99.42 **99.40**	99.89 99.68 99.71 99.78 98.82 96.17 **95.80**	99.66 99.45 99.44 99.72 98.88 97.23 **97.08**	99.66 99.45 99.42 99.72 98.90 97.28 **97.13**	99.89 99.68 99.71 99.78 98.82 96.17 **95.80**	98.63 97.36 97.08 98.61 **92.20** 80.11 79.82	96.76 95.38 95.20 97.88 **93.49** 87.71 87.29
**CrCL 10 mL/min** LD 300 mg, MD 160 mg q 24 h (Nation) LD 300 mg, MD 150 mg q 24 h (Siriraj and EMA) LD 300 mg, MD 60 mg q 24 h (FDA) LD 300 mg, 50 mg q 24 h (Our study) LD 300 mg, MD 50 mg q 48 h (Our study)	160 mg 150 mg 60 mg 50 mg 25 mg	100 100 99.99 99.99 **100**	100 99.99 99.85 99.68 **99.73**	99.97 99.96 99.60 99.43 **99.41**	99.97 99.96 99.60 99.43 **99.41**	100 100 99.85 99.68 **99.73**	99.99 99.91 97.61 96.25 **96.04**	99.65 99.57 96.42 95.35 **94.95**

LD, loading dose (in mg colistin base activity), MD, maintenance dose (in mg colistin base activity), EMA, European Medicines Agency, FDA, Food and Drug Administration of United States, Nation et al.: The research from Nation et al. our study: the proposed regimens in our study. The bold font shows the PTA achieved for ≥ 90% at the lowest dose.

**Table 5:** %PTA and %CFR of the studied sitafloxacin regimens for each type of isolate in combinations.

Dosage regimen	Daily dose (mg)	%PTA of MDR-AB and CRAB		%CFR MDR-AB	%CFR CRAB	%PTA of CoR-AB		%CFR CoR-AB
		MIC_50_ (0.5 mg/L)	MIC_90_ (1 mg/L)			MIC_50_ (0.25 mg/L)	MIC_90_ (1 mg/L)	
**CrCL 90 mL/min** 750 mg q 12 h (Our study) 500 mg q 12 h (Our study) 400 mg q 12 h (Our study) 375 mg q 12 h (Our study) 200 mg q 12 h (Our study) 175 mg q 12 h (Our study) 100 mg q 12 h (Manufacturer) 100 mg q 24 h (Manufacturer) 50 mg q 12 h (Manufacturer)	1,500 mg 1,000 mg 800 mg 750 mg 400 mg 350 mg 200 mg 100 mg 100 mg	100 99.15 **94.14** 91.29 16.79 6.61 0.02 0 0	**91.25** 43.48 16.4 10.95 0.01 0 0 0 0	93.82 47.60 73.79 71.28 34.85 28.91 12.27 6.66 6.64	93.04 47.03 73.20 70.70 34.28 28.36 11.99 6.44 6.43	100 100 100 99.99 94.47 86.99 16.94 0.0 0.03	**91.25** 43.48 16.46 10.95 0.01 0 0 0 0	**97.12** 78.84 88.95 87.97 72.51 67.72 36.54 18.06 18.04
**CrCL 50 mL/min** 500 mg q 12 h (Our study) 425 mg q 12 h (Our study) 400 mg q12 h (Our study) 225 mg q12 h (Our study) 200 mg q 12 h (Our study) 150 mg q12 h (Our study) 125 mg q 12 h (Our study) 100 mg q 12 h (Manufacturer) 100 mg q 24 h (Manufacturer) 50 mg q 12 h (Manufacturer)	1,000 mg 850 mg 800 mg 450 mg 400 mg 300 mg 250 mg 200 mg 100 mg 100 mg	100 100 99.99 **94.83** 88.82 55.24 29.35 8.47 0 0	98.2 **92.32** 88.93 17.78 8.11 0.87 0.09 0.02 0 0	96.06 94.12 93.22 74.40 69.54 53.00 41.08 29.97 10.56 10.49	95.34 93.34 92.43 73.81 68.98 52.43 40.50 29.42 10.31 10.24	100 100 100 100 100 99.6 **97.92** 88.22 8.94 8.54	98.2 **92.32** 88.93 17.78 8.11 0.87 0.09 0.02 0 0	98.21 97.26 **96.85** 89.19 87.31 81.00 76.01 68.54 32.42 32.25
**CrCL 30 mL/min** 500 mg q 24 h (Our study) 400 mg q 24 h (Our study) 300 mg q 24 h (Our study) 250 mg q 24 h (Our study) 200 mg q 24 h (Our study) 150 mg q 24 h (Our study) 125 mg q 24 h (Our study) 100 mg q 24 h (Our study) 50 mg q 24 h (Manufacturer)	500 mg 400 mg 300 mg 250 mg 200 mg 150 mg 125 mg 100 mg 50 mg	100 99.88 97.82 **91.50** 70.23 29.79 10.87 1.96 0	**91.31** 70.16 30.58 11.53 2.27 0.06 0.01 0 0	93.84 88.63 78.64 71.50 59.99 41.27 31.58 23.70 8.72	93.06 87.88 78.01 70.93 59.43 40.69 31.02 23.20 8.49	100 100 100 100 99.88 97.94 **91.07** 70.7 2.03	**91.31** 70.16 30.58 11.53 2.27 0.06 0.01 0 0	97.13 94.92 **90.86** 88.06 83.69 76.09 70.13 60.13 26.91
**CrCL 10 mL/min** 500 mg q 48 h (Our study) 350 mg q 48 h (Our study) 325 mg q 48 h (Our study) 300 mg q 48 h (Our study) 200 mg q 48 h (Our study) 175 mg q 48 h (Our study) 150 mg q 48 h (Our study) 100 mg q 48 h (Our Study) 50 mg q 24 h (Our study) 75 mg q 48 h (Our study) 50 mg q 48 h (Manufacturer)	250 mg 175 mg 162.5 mg 150 mg 100 mg 87.5 mg 75 mg 50 mg 50 mg 37.5 mg 25 mg	100 99.99 100 99.99 97.79 **93.68** 83.58 30.01 30.09 6.04 0.13	99.78 94.11 **90.01** 83.5 29.77 14.52 5.44 0.11 0.07 0 0	97.52 94.61 93.49 91.85 78.44 73.15 66.62 41.37 41.45 27.93 14.84	96.94 93.84 92.70 91.07 77.81 72.57 66.06 40.79 40.87 27.39 14.51	100 100 100 100 100 100 99.98 97.88 **98.17** 83.22 29.51	99.78 94.11 **90.01** 83.5 29.77 14.52 5.44 0.11 0.07 0 0	99.10 97.48 96.97 96.26 **90.78** 88.69 86.20 76.10 76.23 66.05 42.27

Our study: the proposed regimens in our study, manufacturer: the recommended dosage regimens from the manufacturer of sitafloxacin. The bold font shows the PTA achieved for ≥ 90% at the lowest dose.

## Discussion

The infection caused by drug-resistant *A. baumannii* is a serious problem, especially MDR-AB and CRAB. Because of the various types of antibiotics that the pathogen resists, the choice of drug is limited. This study focuses on the use of colistin, which is known as the last resort for gram-negative bacteria, especially the drug-resistant pathogen. Moreover, sitafloxacin was chosen based on the good activity from the previous study (Rodjun et al., 2020). This is the first study to explore colistin dosage regimens based on the new pharmacokinetic/pharmacodynamic (PK/PD) index of *A. baumannii*. To date, the recommended colistin dosage regimen aims to achieve the desired C_ss,avg_, especially at 2 mg/L (Garonzik et al., 2011; Nation et al., 2017), formerly the susceptibility breakpoint of gram-negative isolates, including *Acinetobacter* spp. This study used ƒAUC/MIC ≥7.4 as the desired index (Cheah et al., 2015). We ran simulations starting with a loading dose of 300 mg (the same as in the reference dosage regimens) and 450 mg, expecting them to rapidly reach the steady state of colistin. The reference dosage regimens recommended by FDA, EMA, Siriraj, and Nation et al. for the simulation in patients with a CrCL value of ≥90 mL/min cannot achieve the specific index at a PTA of ≥90%; however, our study’s dosage regimens can achieve it with the maintenance dose of 225 mg every 12 h at breakpoint MIC. The results of Jitaree et al., who studied the optimal dosage of colistin against carbapenem-resistant *Klebsiella pneumonia* and carbapenem-resistant *Escherichia coli* (Jitaree et al., 2019), were consistent with ours. In a patient who has a normal renal function (≥80 mL/min), one cannot use the reference dosage regimen to achieve the specific index (ƒAUC/MIC ≥25) when using the breakpoint as a desired MIC. Therefore, the usual colistin dosage regimens cannot achieve the specific index of each important gram-negative isolate in a patient who has normal renal function. However, the reference dosage regimen can achieve the specific index in a patient who has renal impairment (≤50 mL/min). Interestingly, our new dosage regimens (150 mg, 75 mg, and 50 mg every 24 h for CrCL value of 50, 30, and 10 mL/min, respectively), which are lower than the lowest reference dose (114 mg every 12 h, 150 mg every 24 h, and 60 mg every 24 h for CrCL value of 50, 30, and 10 mL/min, respectively), can achieve the target. To the best of our knowledge, the most common adverse event observed with colistin is dose-dependent nephrotoxicity (Nation et al., 2014; Eljaaly et al., 2021). A lower dose should be considered to reduce the risk of nephrotoxicity in a patient who has renal function impairment. However, patients with good renal function seem to be using the higher than usual. The other adverse reaction is neurotoxicity (Spapen et al., 2011). Though colistin is the last resort for the treatment of infection by a drug-resistant organism, the rate of colistin resistance is currently a problem (Cai et al., 2012). The reference dosage regimen for colistin cannot be used against the colistin-resistant *A. baumannii* in this study (MIC ≥2 mg/L), especially in a patient who has CrCL ≥50 mL/min. Therefore, combination regimens should be considered. However, the international consensus guidelines (Tsuji et al., 2019) for the optimal use of polymyxins recommended the use of a C_ss,avg_ of 2 mg/L instead of the new index because of the differences in the protein binding of mice and humans. However, our study used a protein binding profile from Nation et al. (2017), who ran simulations in a critically ill patient. The PK/PD index at a C_ss,avg_ of 2 mg/L did not depend on the variation in MIC values, which might complicate the choice of optimal doses. The new PK/PD index used in this study can be applied to any MIC value. Moreover, our PK/PD index is specific for *A. baumannii* (Cheah et al., 2015).

Sitafloxacin, the fluoroquinolone antibiotic, demonstrates the concentration-dependent killing effect. The lower concentration showed less bacteriological efficacy (Tanigawara et al., 2013). The reference regimen for sitafloxacin monotherapy (50–100 mg every 12–48 h) cannot achieve the specific index in each CrCL at a PTA of ≥90%, while our study’s regimens can (≥175 mg every 48 h). We used the same PK/PD index as Tanigawara et al., who were the only authors to study the PK/PD index of sitafloxacin, ƒAUC/MIC ≥30 (Huang et al., 2015), but there were differences between their MIC values and ours. The lowest MIC from the study by Tanigawara et al. was ≤0.025 mg/L, which was less than the focus MIC value of this study, ≥0.5 mg/L, which led them to a lower recommended dose than the one in this study. In fact, the approved indications of sitafloxacin are likely to be a mild infection or community-acquired infection (Pharmaceuticals and Medical Devices Agency, n.d.), which is why the manufacturer-recommended dose seems to be low.

The colistin-sitafloxacin combination led to a greater opportunity to explore the optimal dosage regimen to achieve a PTA of 90% based on the specific PK/PD index. Because the MIC50/90 of CoR-AB (2 mg/L) was reduced to at least the intermediate breakpoint (Clinical and Laboratory Standards Institute, 2022) (≤2 mg/L), this study could determine the dosage regimen for all CrCL values. Although the MIC of sitafloxacin was also reduced, the dosage regimens that achieve a PTA of 90% are still higher than the reference dosage regimens. For example, 50 mg of sitafloxacin every 24 h was obtained for a patient with a CrCL value of 10 mL/min, while the recommended dose is 50 mg every 48 h. To our knowledge, fluoroquinolones are concentration-dependent antibiotics (Lode et al., 1998) and sitafloxacin is a fluoroquinolone antibiotic agent (Sun et al., 2021). Therefore, higher doses can have greater efficacy in isolate eradication. Saito et al. (2008) showed greater efficacy of sitafloxacin in a higher dose, which is consistent with our result that a higher dose achieved a higher PTA percentage. The point of concern is the possibility of a dose-dependent adverse drug reaction. Feldman et al. used sitafloxacin at a high dose (400 mg intravenously once daily) for the treatment of hospitalized patients with pneumonia and showed a 5% rate of drug-related adverse events but no severe reactions (Feldman et al., 2001). In a previous report, the dose-dependent prolongation of QTcF occurred after the administration of supratherapeutic dosages of sitafloxacin of 400, 600, or 800 mg twice daily to healthy volunteers (mean change in the QTcF interval of 0, 6, and 10 ms, respectively) (Keating, 2011). The highest recommended dose of sitafloxacin from this study for combination therapy in a patient with a CrCL value of 90 mL/min was 200–750 mg every 12 h, which might increase the QTcF. However, no reports of serious adverse reactions or adverse events based on recent data on sitafloxacin were observed.

This study explored all dosage regimens in a patient with various CrCL values, focusing on the one that achieved a PTA of 90% for the MIC90 of each agent in combination therapy. A colistin dose lower than the usual one seems to have led to less nephrotoxicity, while a dose of sitafloxacin appears to be higher than the usual regimen. A patient who is administered a high dose requires close monitoring.

An optimal CFR of ≥90% was used in this study. Some dosage regimens were different from the dosage regimen for a PTA of ≥90% because of the MIC distribution. The dose of sitafloxacin that achieved a CFR of ≥90% was lower than that which achieved a PTA of 90% in each CrCL value. The major MIC distribution of sitafloxacin in our study was lower than 0.5 mg/L (Rodjun et al., 2020), which increases the probability of achieving the desired CFR. The doses of colistin required to achieve a CFR of ≥90% in MDR-AB and CRAB for each CrCL were the same because most of the isolates in these two groups overlapped. Thus, the opportunity to achieve the desired CFR was dependent on the MIC distribution.

Our study has several limitations. First, this study used the PK/PD index from the thigh infection in the mouse model. For the treatment of other types of infections, another specific index should be considered. Drug-resistant *A. baumannii*, including MDR-AB, CRAB, and CoR-AB, could be treated by combination therapy because it increases the probability of achieving the specific target. Second, most of the recommended doses from this study were different from the reference dosage regimens. Therefore, close monitoring of clinical efficacy and toxicity is necessary. Third, our dosing recommendation used two population pharmacokinetics of two agents (colistin and sitafloxacin) to simulate. Therefore, our dosing recommendation for colistin and sitafloxacin could be used only in patients with similar characteristics to this study. According to the population pharmacokinetic model, for instance, there is a difference in the APACHE II score, the comorbid condition, the CrCL, the volume of distribution, and the drug clearance in critically ill patients compared to the healthy population. These parameters affect the plasma colistin level, which is important to reach the desired target in critically ill patients. Although the population pharmacokinetic analysis of sitafloxacin was based on the study on non-critically ill patients, the oral form of this drug should be considered for absorption in critically ill patients. However, we believe that sitafloxacin might have poor absorption as critically ill patients are hemodynamically unstable. The low plasma concentration might have occurred and cannot reach the target. Fourth, drug interaction should be considered because sitafloxacin (fluoroquinolone group) could reduce absorption when concomitant with antacids, ferrous sulfate, and other metallic cation-containing compounds (Deppermann and Lode, 1993) (this study simulates based on the fasted state of patient). Finally, this study used the PD index of efficacy. Thus, we cannot predict the resistant situation. However, the high dose of sitafloxacin should not develop pathogen resistance. The low dose of colistin in the combination therapy was against the lower MIC than usual. They should not develop pathogen resistance either. Currently, the problem with using colistin and sitafloxacin in combination is that there are no data that relate to the clinical outcome in terms of resistant genes. Although this study uses data from the most recent publications, further study is needed to confirm the results of this simulation study.

## Data availability statement

The original contributions presented in the study are included in the article/Supplementary material, further inquiries can be directed to the corresponding author.

## Ethics statement

This study was approved by the Ethics Committee of the Faculty of Dentistry/Faculty of Pharmacy, Mahidol University (COE.No.MU-DT/PY-IRB2020/001.1501).

## Author contributions

VR: Conceptualization, Data curation, Formal analysis, Investigation, Methodology, Project administration, Writing – original draft, Writing – review & editing. PM: Conceptualization, Project administration, Supervision, Validation, Writing – review & editing. JH: Methodology, Resources, Writing – review & editing. KJ: Methodology, Writing – review & editing. WN: Methodology, Writing – review & editing.
