# Supplementary material for: Pharmacokinetic/pharmacodynamic (PK/PD) simulation for dosage optimization of colistin and sitafloxacin, alone and in combination, against carbapenem-, multidrug-, and colistin-resistant Acinetobacter baumannii
Source: Front Microbiol. 2023 Nov 30;14:1275909. doi: 10.3389/fmicb.2023.1275909 (PMC10720588; doi:10.3389/fmicb.2023.1275909)
Supplement: Supplementary file 1 [file Table_1.DOCX]

Supplementary Material

**Table S1. %PTA and %CFR of the study colistin regimens for each type of isolates (all regimens follow the loading dose of 300 mg)**

| MIC (mg/L) | MDR-AB and CRAB | | | | CoR-AB | |
| --- | --- | --- | --- | --- | --- | --- |
|  | %PTA | | %CFR MDR-AB | %CFR CRAB | %PTA | %CFR |
|  | MIC_50_ (2 mg/L) | MIC_90_ (4 mg/L) |  |  | MIC_50/90_ (8 mg/L) |  |
| **CrCL 90 ml/min**  300 mg q 12 h (our study)  150 mg q 8 h (Siriraj)  180 mg q 12 h (Nation et al)  150 mg q 12 h (Siriraj, EMA, FDA)  100 mg q 12 h (our study)  75 mg q 12 h (our study) | **94.06**  82.97  86.87  82.55  67.16  51.48 | 81.82  50.17  62.39  52.70  28.52  12.59 | **91.43**  80.48  84.11  80.38  68.48  57.08 | **91.57**  80.79  84.36  80.67  68.83  57.42 | 52.24  10.44  22.10  13.36  2.20  0.31 | 63.03  28.89  39.82  31.36  14.85  6.28 |
| **CrCL 50 ml/min**  300 mg q 12 h (our study)  150 mg q 8 h (our study)  180 mg q 12 h (our study)  150 mg q 12 h (Siriraj, EMA, FDA)  122.5 mg q 12 h (Nation et al)  114 mg q 12 h (FDA)  100 mg q 12 h (our study)  75 mg q 12 h (our study)  50 mg q 12 h (our study) | 99.28  96.32  97.62  95.83  93.44  **92.66**  89.57  80.51  56.82 | **96.2**  78.4  87.27  80.42  70.15  66.03  56.72  34.59  8.72 | 97.29  91.34  93.72  **91.42**  88.77  87.73  85.11  78.17  62.65 | 97.33  91.57  93.86  **91.62**  89.03  88.02  85.43  78.54  63.04 | 80.25  28.57  49.35  34.64  20.57  15.54  9.11  1.58  0.04 | 83.82  50.35  63.80  53.95  42.90  38.78  31.52  17.55  4.28 |
| **CrCL 30 ml/min** 300 mg q 12 h (our study)  150 mg q 8 h (our study)  150 mg q 12 h (our study)  125 mg q 12 h (EMA)  100 mg q 12 h (Siriraj)  97.5 mg q 12 h (Nation et al)  150 mg q 24 h (FDA)  100 mg q 24 h (our study)  75 mg q 24 h(our study)  60 mg q 24 h (our study)  50 mg q 24 h (our study) | 99.93  99.11  99.36  98.63  97.46  97.09  98.62  96.11  **92.01**  86.52  80.14 | 99.28  90.5  **93.1**  88.52  77.8  75.81  92.19  79.95  66.9  53.39  41.58 | 98.90  94.65  95.49  93.84  91.52  91.10  95.83  **91.99**  88.05  83.37  78.65 | 98.91  94.79  97.77  94.00  91.77  91.36  95.92  **92.18**  88.31  83.69  78.99 | **93.39**  47.58  56.53  40.12  21.41  19.48  66.74  39.59  24.56  12.88  7.42 | **92.87**  64.39  69.69  60.18  46.99  45.20  75.14  56.24  43.15  31.53  23.44 |
| **CrCL 10 ml/min**  160 mg q 24 h (Nation et al)  150 mg q 24 h (Siriraj, EMA)  100 mg q 24 h (our study)  75 mg q 24 h (our study)  60 mg q 24 h (FDA) | 99.97  99.98  99.71  **99.02**  86.52 | 99.36  99.06  95.73  **91.17**  53.39 | 98.95  98.76  97.10  **95.29**  83.37 | 98.96  98.78  97.16  **95.40**  83.69 | 92.47  **90.35**  74.29  56.58  12.88 | 92.92  **91.48**  80.34  69.34  31.53 |

**Table S2. %PTA and %CFR of the study sitafloxacin regimens for each type of isolates**

| MIC (mg/L) | MDR-AB and CRAB | | | | CoR-AB | | |
| --- | --- | --- | --- | --- | --- | --- | --- |
|  | %PTA | | %CFR MDR-AB | %CFR CRAB | %PTA | | %CFR |
|  | MIC_50_ (1 mg/L) | MIC_90_ (2 mg/L) |  |  | MIC_50_ (0.5 mg/L) | MIC_90_ (1 mg/L) |  |
| **CrCL 90 ml/min**  1500 mg every 12 h (our study)  1000 mg every 12 h (our study)  800 mg every 12 h (our study)  750 mg every 12 h (our study)  500 mg every 12 h (our study)  400 mg every 12 h (our study)  375 mg every 12 h (our study)  200 mg every 12 h (our study)  100 mg every 12 h (manufacturer)  100 mg every 24 h (manufacturer)  50 mg every 12 h (manufacturer) | 99.99  99.15  94.77  **91.25**  43.48  16.46  10.95  0.01  0  0  0 | **90.94**  43.79  16.79  11.15  0.3  0.02  0  0  0  0  0 | **95.31**  86.60  80.07  77.58  54.76  41.48  38.22  10.67  3.48  2.66  2.66 | 95.29  86.78  80.35  77.87  54.88  41.49  38.21  10.77  3.87  3.12  3.12 | 100  100  100  100  99.15  94.14  **91.29**  16.79  0.02  0  0 | 99.99  99.15  94.77  **91.25**  43.48  16.46  10.95  0.01  0  0  0 | 95.18  **92.48**  89.90  88.57  73.25  62.85  59.93  23.19  8.56  6.85  6.85 |
| **CrCL 50 ml/min**  1000 mg every 12 h (our study)  825 mg every 12 h (our study)  800 mg every 12 h (our study)  750 mg every 12 h (our study)  500 mg every 12 h (our study)  425 mg every 12 h (our study)  400 mg every 12 h (our study)  225 mg every 12 h (our study)  200 mg every 12 h (our study)  100 mg every 12 h (manufacturer)  100 mg every 24 h (manufacturer)  50 mg every 12 h (manufacturer) | 100  100  99.98  99.99  98.2  **92.32**  88.93  17.78  8.11  0.02  0  0 | 98.09  **90.04**  88.39  83.11  30.24  12.79  9  0.03  0.02  0  0  0 | 97.06  95.16  94.82  **93.83**  83.86  78.33  76.21  42.26  36.24  7.97  3.24  3.23 | 97.01  95.15  94.81  93.84  84.10  78.61  76.49  42.28  36.22  8.11  3.65  3.65 | 100  100  100  100  100  100  99.99  **94.83**  88.82  8.47  0  0.01 | 100  100  99.98  99.99  98.2  **92.32**  88.93  17.78  8.11  0.02  0  0 | 95.96  95.14  95.01  94.69  **91.56**  88.97  87.77  63.56  57.98  18.93  7.81  7.77 |
| **CrCL 30 ml/min** 1000 mg every 24 h (our study)  800 mg every 24 h (our study)  750 mg every 24 h (our study)  500 mg every 24 h (our study)  400 mg every 24 h (our study)  300 mg every 24 h (our study)  250 mg every 24 h (our study)  200 mg every 24 h (our study)  150 mg every 24 h (our study)  100 mg every 24 h (our study)  50 mg every 24 h (manufacturer) | 99.98  99.87  99.74  **91.31**  70.16  30.58  11.53  2.27  0.06  0  0 | **91.45**  70.3  61.5  11.29  1.63  0.07  0  0  0  0  0 | 95.41  **91.50**  89.90  77.63  66.78  48.73  38.53  28.05  14.74  5.52  2.98 | 95.39  91.56  90.01  77.92  67.01  48.79  38.53  28.04  14.80  5.72  3.42 | 100  100  100  100  99.88  97.82  **91.50**  70.23  29.79  1.96  0 | 99.98  99.87  99.74  **91.31**  70.16  30.58  11.53  2.27  0.06  0  0 | 95.21  93.98  **93.50**  88.60  81.70  68.75  60.20  47.99  29.27  14.42  7.16 |
| **CrCL 10 ml/min**  750 mg every 48 h (our study)  700 mg every 48 h (our study)  675 mg every 48 h (our study)  500 mg every 48 h (our study)  350 mg every 48 h (our study)  325 mg every 48 h (our study)  300 mg every 48 h (our study)  200 mg every 48 h (our study)  175 mg every 48 h (our study)  150 mg every 48 h (our study)  100 mg every 48 h (our study)  50 mg every 24 h (our study)  75 mg every 48 h (our study)  50 mg every 48 h (manufacturer) | 100  100  99.99  99.78  94.11  **90.01**  83.5  29.77  14.52  5.44  0.11  0.07  0  0 | 96.24  94.35  **91.9**  62.19  15.22  9.61  5.38  0.06  0.02  0  0  0  0  0 | 95.68  96.12  95.68  93.55  89.62  78.51  85.96  68.49  44.94  54.85  29.38  29.43  17.39  9.78 | 96.48  96.00  95.52  90.15  79.80  76.78  73.51  48.43  40.50  33.46  14.89  14.90  7.25  4.20 | 100  100  100  100  99.99  100  99.99  97.79  **93.68**  83.58  30.01  30.09  6.04  0.13 | 100  100  99.99  99.78  94.11  **90.01**  83.5  29.77  14.52  5.44  0.11  0.07  0  0 | 95.68  95.97  95.49  **93.55**  89.62  77.07  85.96  68.49  40.50  54.85  29.38  29.43  17.39  9.78 |

**
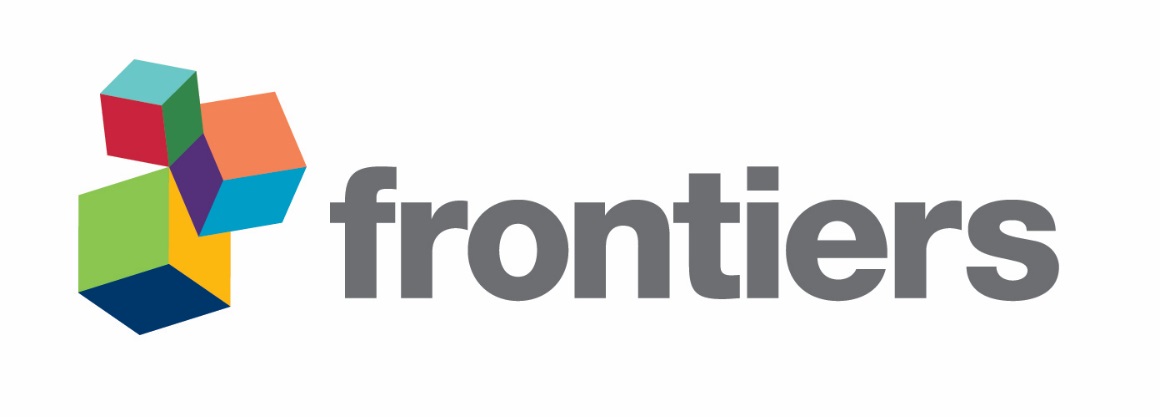
**
